# Cervical HPV infection in Guangzhou, China: an epidemiological study of 198,111 women from 2015 to 2021

**DOI:** 10.1080/22221751.2023.2176009

**Published:** 2023-02-15

**Authors:** Xiaohan Yang, Yuanyuan Li, Yuan Tang, Zhiyu Li, Sanfeng Wang, Xiping Luo, Tianwen He, Aihua Yin, Mingyong Luo

**Affiliations:** a Medical Genetic Center, Guangdong Women and Children Hospital, Guangzhou, People’s Republic of China; b Guangzhou Institute of Respiratory Health, the First Affiliated Hospital of Guangzhou Medical University, Guangzhou Medical University, Guangzhou, People’s Republic of China; c Guangdong Women and Children's Hospital, Guangzhou Medical University, Guangzhou, People’s Republic of China; d Department of Gynecology and Obstetrics, Guangdong Women and Children Hospital, Guangzhou, People’s Republic of China; e Department of Clinical Laboratory, Guangdong Women and Children Hospital, Guangzhou, People’s Republic of China

**Keywords:** Human papillomavirus, epidemiology, normal cervix, cervical lesions

## Abstract

Persistent high-risk human papillomavirus (HPV) infection is the pivotal cause of cervical carcinogenesis. HPV types distribution varies greatly by region, and its long-term changes of prevalence remain to be fully characterized in China. Here, the largest population of 198,111 consecutive women who underwent routine cervical screening were investigated from 2015 to 2021 in Guangzhou, south China. The results showed that the overall HPV prevalence was 21.66% (42,911/198,111), and the annual prevalence increased significantly from 2015 to 2021 (*p* < 0.001). HPV52, 16, 58, CP8304, 51, 53, 39, and 68 were the most prevalent HPV types. The relative HPV-positive rate correlated positively with the progression of cervical intraepithelial neoplasia (*p* < 0.001); HPV16 was the predominant carcinogenic type, followed by HPV52 and HPV18. HPV infections were significantly age-specific, and 26.51% (11,375/42,911) of cases were caused by multiple HPV types. In addition, HPV infections typically cleared over a median time of 16 (interquartile range 9–31) months, and the clearance of HPV16 was significantly faster than that of other types (*p* < 0.001). These findings may serve as a guide for local governments to evaluate HPV vaccination and cervical cancer prevention strategies in south China.

## Introduction

Cervical cancer (CC) is the fourth most common malignancy in women. Approximately 604,000 CC cases and 342,000 deaths, equivalent to 7.7% of all cancer deaths, were reported worldwide in 2020 by the International Agency for Research on Cancer (IARC) [1]. In forty-two low-resource countries, CC is number one cause of cancer deaths among women [1,2]. Persistent specific human papillomavirus (HPV) infection is identified as the main cause of cervical intraepithelial lesions and occurs in 84% of individuals with CC [3].

HPV is typically divided into “low-risk” and “high-risk” types [4], and 12 high-risk types (HPV16, 18, 31, 33, 35, 39, 45, 51, 52, 56, 58, and 59) have been designated as group 1 carcinogens by the IARC [5]. HPV16 and HPV18, the most commonly noted types, jointly cause the majority of CC and its precursors [6]. Moreover, HPV is also responsible for other premalignant and malignant lesions, including penile, vaginal, anal, vulvar, oropharyngeal, and pulmonary cancers, as well as condyloma acuminata (CA) [7–9].

Vaccination against HPV16 and 18, and some other high-risk types has the potential to prevent HPV-related infection by up to 90% [6,10]. Moreover, cytological screening and HPV testing have been used for clinical comprehensive CC control. The treatment of cervical lesions detected through the Pap-smear screening of cervical cells has been the paradigm of secondary prevention for several decades and undoubtedly has greatly reduced the morbidity of CC [7]. As screening based on HPV protects against future cervical lesions better than cervical cytology [11,12], and therefore, HPV testing every 5 years has been recommended by the WHO [13]. However, owing to the lack of efficacious CC screening programs, high costs, policy limits of HPV vaccination, limited healthcare services in many (mostly) developing countries, and these vaccines being considered low-priority, CC remains one of the most frequent malignancies affecting females.

As the main contributor to the global cancer burden, China accounted for 18.7% of the overall morbidity and 15.3% of the mortality associated with CC [3]. Given the large population of this country with low vaccine coverage, its vast landmass, and uneven levels of economic development, HPV epidemiological features vary geographically and temporally and its associated cancer burden across regions in China remain to be fully characterized [14–17]. A detailed understanding of HPV epidemics in specific regions and their association with cancer may help in enhancing vaccination strategies and CC screening. Therefore, we sought to perform a large and long-term study to identify the HPV burden in women with different cervical pathology status in Guangzhou, the largest city in south China, to guide the HPV-targeted vaccination strategies and CC prevention programs.

## Materials and methods

### Study subjects

The study population constituted women who underwent routine cervical exams at Guangdong Women and Children Hospital between 2015 and 2021 in Guangzhou, China. All females underwent genotype testing of HPV and cervical cytological evaluations combined with colposcopy. The exclusion criteria were pregnancy, a total hysterectomy, systemic infection or autoimmune diseases, surgery for uterine diseases within 3 days, or other cancers. Ethics approval was obtained from the Ethics Committee of the Guangdong Women and Children Hospital (approval number 202301006).

### Histopathological diagnosis

Patients with cervical abnormalities, based on cytological screening cytological screening (ThinPrep cytological test, TCT) and colposcopy, underwent histopathological examination for a final diagnosis as per the criteria of Bethesda 2014 [18]. Pathological results were characterized as follows: (a) a normal cervix; (b) inflammation; (c) a low-grade squamous intraepithelial lesion (LSIL), which describes those with cervical intraepithelial neoplasia grade (CIN) I; (d) a high-grade squamous intraepithelial lesion (HSIL), which describes those with CIN II or CIN III; (e) atypical squamous cells of undetermined significance or inability to exclude HSIL (ASC-US/-H); (f) invasive cervical carcinoma (ICC); (g) condyloma acuminate (CA); and (h) others, which include those who underwent surgery or who were treated for hysteromyoma, among other cases.

### HPV genotyping

HPV deoxyribonucleic acid (DNA) typing was performed using an HPV genotyping kit (Kaipu Biotechnology, China), which was based on DNA amplification with HPV L1 consensus polymerase chain reaction primers and the flow-through hybridization technique. It can recognize 21 common types (15 high-risk and six low-risk). All protocols were performed according to supplier's manual, as described previously [19,20].

### Time to HPV clearance

HPV clearance was defined as one getting at least two consecutive HPV-negative results after being tested positive. The HPV-positive women who received HPV DNA testing at least once a year within the subsequent 5 years after the initial visit were enrolled in the cohort of HPV clearance, excluding HPV infections confirmed before the initial visit and those who had one or more HPV types and obtained a new and different HPV type later. The estimated time to HPV clearance in HPV-positive women was depicted as a Kaplan–Meier plot, and the hazard ratios (HRs) and 95% confidence intervals (CIs) for the time to HPV clearance were calculated using a Cox proportional-hazards model.

### Statistical analysis

SPSS 25.0 (Chicago, USA) was used for statistical analysis. The quantitative data are presented as the median and interquartile range (IQR), and Kruskal–Wallis/Dunn’s test were used for comparisons between groups. The categorical variables are described as percentages, and the χ^2^ test was used to evaluate the significant differences. The linear regression models with gamma value were used to assess the changes of prevalence in different diagnosis groups and over the calendar year. Chord diagrams were constructed using HPV type co-infection data. Moreover, to quantify the age patterns of different diagnoses, we also created a heatmap of the proportions of age groups identified in each group. *p* < 0.05 was considered statistically significant.

## Results

### Demographic and clinical characteristics

**Figure 1.**
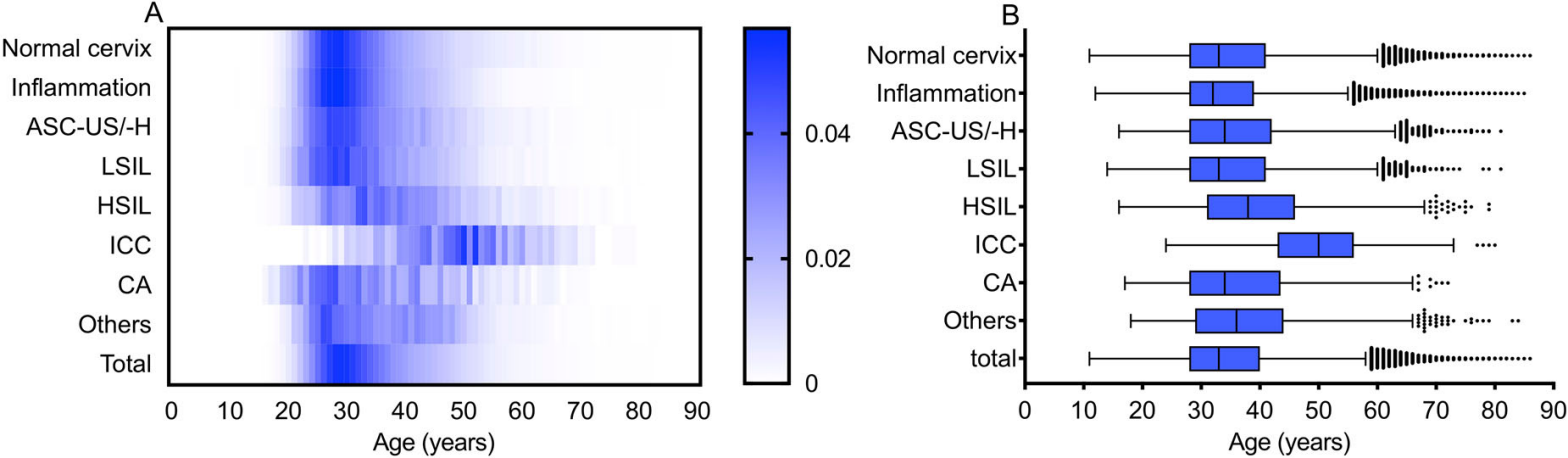
Age distribution according to diagnosis in Guangzhou, China. (A) Heatmap of age distribution according to diagnosis, standardized by the number of diagnosis groups. Blue square shows the percentage of positive diagnoses. (B) Age distribution (median, interquartile range) of cases according to the diagnosis. LSIL, low-grade squamous lesion; HSIL, high-grade squamous lesion; ICC, invasive cervical carcinoma; CA, condyloma acuminate, ASC-US/-H, atypical squamous cells of undetermined significance or HSIL not excluded.

In total, 198,111 females were enrolled, with a median age of 33 (IQR 28–40) years. Among them, 53,439 (26.97%) had a normal cervix, 112,999 (57.04%) were diagnosed with inflammation, 12,251 (6.18%) were diagnosed with ASC-US/-H, 8108 (4.09%) were diagnosed with an LSIL, 3243 (1.64%) were diagnosed with an HSIL, 799 (0.40%) were diagnosed with ICC, 1257 (0.63%) were diagnosed with CA, and the remaining 6015 (3.04%) cases showed other clinical features. As shown in Figure 1, significant differences in age were observed among the different diagnoses (H = 2605.410, *p *< 0.001). The median age was 50 (43–56) years in the ICC group, which was significantly higher than that in the other groups (all *p *< 0.001); in contrast, the median age was 32 (28–39) years in the inflammation group (all *p *< 0.001), which was lower than that in the other groups. However, no significant difference was observed between the LSIL and normal cervix groups (*p* > 0.05).

### HPV prevalence and type distribution

As shown in Table 1, the overall HPV-positivity rate was 21.66% (42,911/198,111); 18.63% of patients were identified as having high-risk HPV infection, whereas 5.46% were infected with low-risk types. HPV52 (11,093, 5.60%) was the most common type, followed by HPV16 (5707, 2.88%), 58 (5132, 2.59%), CP8304 (3832, 1.93%), 51 (3816, 1.93%), 53 (3761, 1.90%), 39 (3725, 1.88%), and 68 (3062, 1.55%). Regarding different diagnoses, the HPV prevalence was 16.01% (8557/53,439), 15.10% (17,066/112,999), 48.58% (5952/12,251), 78.91% (6398/8108), 84.06% (2726/3243), 84.48% (675/799), 33.65% (423/1257), and 18.52% (1114/6015) among the normal cervix, inflammation, ASC-US/-H, LSIL, HSIL, ICC, CA, and other groups, respectively.

**Table 1.** Distribution of HPV types according to final diagnostic status among the 198111 females.

HPV type	Total	Normal cervix	Inflammation	ASC-US/-H	LSIL	HSIL	ICC	CA	Others
Number of cases	198111	53439 (26.97)	112999 (57.04)	12251 (6.18)	8108 (4.09)	3243 (1.64)	799 (0.40)	1257 (0.63)	6015 (3.04)
Positive	42911 (21.66)	8557 (16.01)	17066 (15.10)***	5952 (48.58)***	6398 (78.91)***	2726 (84.06)***	675 (84.48)***	423 (33.65)***	1114 (18.52)***
Low-risk HPV									
Positive	10826 (5.46)	2276 (4.26)	4574 (4.05)*	1366 (11.15)***	1485 (18.32)***	451 (13.91)***	105 (13.14)***	265 (21.08)***	304 (5.05)**
HPV6	1568 (0.79)	294 (0.55)	563 (0.50)	198 (1.62)***	267 (3.29)***	65 (2.00)***	12 (1.50)**	142 (11.30)***	27 (0.45)
HPV11	911 (0.46)	153 (0.29)	327 (0.29)	138 (1.13)***	167 (2.06)***	20 (0.62)**	2 (0.25)	88 (7.00)***	16 (0.27)
HPV42	1975 (1.00)	395 (0.74)	841 (0.74)	292 (2.38)***	261 (3.22)***	100 (3.08)***	21 (2.63)***	18 (1.43)*	47 (0.78)
HPV43	1480 (0.75)	319 (0.60)	648 (0.57)	187 (1.53)***	216 (2.66)***	46 (1.42)***	7 (0.88)	14 (1.11)	43 (0.71)
HPV44	1933 (0.98)	469 (0.88)	857 (0.76)*	219 (1.79)***	198 (2.44)***	86 (2.65)***	19 (2.38)***	18 (1.43)	67 (1.11)
CP8304	3832 (1.93)	814 (1.52)	1623 (1.44)	466 (3.80)***	554 (6.83)***	175 (5.40)***	48 (6.01)***	26 (2.07)	126 (2.09) **
High-risk HPV									
Positive	36910 (18.63)	7149 (13.38)	14094 (12.47)***	5319 (43.42)***	5942 (73.29)***	2591 (79.90)***	617 (77.22)***	296 (23.55)***	902 (15.00)***
HPV16	5707 (2.88)	822 (1.54)	1655 (1.46)	825 (6.73)***	1022 (12.60)***	918 (28.31)***	297 (37.17)***	61 (4.85)***	107 (1.78)
HPV18	2331 (1.18)	376 (0.70)	801 (0.71)	375 (3.06)***	444 (5.48)***	175 (5.40)***	67 (8.39)***	24 (1.91)	69 (1.15)***
HPV31	1223 (0.62)	236 (0.44)	459 (0.41)	198 (1.62)***	181 (2.23)***	97 (2.99)***	22 (2.75)***	7 (0.56)	23 (0.38)
HPV33	1690 (0.85)	284 (0.53)	512 (0.45)*	249 (2.03)***	319 (3.93)***	238 (7.34)***	39 (4.88)***	12 (0.95)*	37 (0.62)
HPV35	589 (0.30)	111 (0.21)	199 (0.18)	96 (0.78)***	122 (1.50)***	28 (0.86)***	8 (1.00)***	5 (0.40)	20 (0.33)*
HPV39	3725 (1.88)	774 (1.45)	1525 (1.35)	513 (4.19)***	600 (7.40)***	161 (4.96)***	32 (4.01)***	34 (2.70)***	86 (1.43)
HPV45	570 (0.29)	124 (0.23)	220 (0.19)	63 (0.51)***	85 (1.05)***	38 (1.17)***	14 (1.75)***	11 (0.88)***	15 (0.25)
HPV51	3816 (1.93)	722 (1.35)	1414 (1.25)	559 (4.56)***	785 (9.68)***	182 (5.61)***	29 (3.63)***	36 (2.86)***	89 (1.48)
HPV52	11093 (5.60)	2130 (3.99)	4333 (3.83)	1703 (13.90)***	1785 (22.02)***	669 (20.63)***	110 (13.77)***	95 (7.56)***	268 (4.46)*
HPV53	3761 (1.90)	786 (1.47)	1409 (1.25)***	530 (4.33)***	688 (8.49)***	176 (5.43)***	33 (4.13)***	44 (3.50)***	95 (1.58)
HPV56	1842 (0.93)	350 (0.65)	611 (0.54)**	256 (2.09)***	443 (5.46)***	94 (2.90)***	14 (1.75)***	25 (1.99)***	49 (0.81)
HPV58	5132 (2.59)	910 (1.70)	1628 (1.44)***	772 (6.30)***	1037 (12.79)***	575 (17.73)***	64 (8.01)***	35 (2.78)**	11 1 (1.85)
HPV59	1245 (0.63)	246 (0.46)	423 (0.37)*	214 (1.75)***	233 (2.87)***	63 (1.94)***	22 (2.75)***	15 (1.19)***	29 (0.48)
HPV66	1658 (0.84)	329 (0.62)	585 (0.52)*	206 (1.68)***	386 (4.76)***	95 (2.93)***	9 (1.13)	16 (1.27)**	32 (0.53)
HPV68	3062 (1.55)	614 (1.15)	1271 (1.12)	451 (3.68)***	463 (5.71)***	135 (4.16)***	21 (2.63)***	21 (1.67)	86 (1.43) *

HPV, human papillomavirus; ASC-US/-H, atypical squamous cells of undetermined significance or cannot excluded HSIL; LSIL, low-grade squamous lesion; HSIL, high-grade squamous lesion; ICC, invasive cervical carcinoma; CA, condyloma acuminate.

Normal cervix is served as test control.

***The significance is at the *p* < 0.001 level.

**The significance is at the *p* < 0.01 level.

*The significance is at the *p* < 0.05 level.

HPV52 (3.99%), HPV58 (1.70%), and HPV16 (1.54%) were the most observed types in normal cervixes, similar to the prevalence of HPV52 (3.83%), HPV58 (1.44%), and HPV16 (1.46%) in the inflammation group. In the abnormal cervix groups (ASC-US/-H, LSIL, HSIL, and ICC), the positivity rates for all high-risk types were significantly higher than those in the normal cervix group (all *p *< 0.001), except for HPV66 in ICC (*p *> 0.05). Specifically, the most prevalent types were HPV52 (13.90%), HPV16 (6.73%), and HPV58 (6.30%) in ASC-US/-H patients; HPV52 (22.02%), HPV58 (12.79%), and HPV16 (12.60%) in LSIL patients; HPV16 (28.31%), HPV52 (20.63%), and HPV58 (17.73%) in HSIL patients; and HPV16 (37.17%), HPV52 (13.77%), and HPV18 (8.39%) in ICC patients (Table 1). Additionally, HPV52 (7.56%) and low-risk HPV6 (11.30%) and HPV11 (7.00%) were the major types in patients with CA. The annual prevalence of HPV showed an obvious increasing trend from 2015 to 2021 (*p *< 0.001). In detail, the prevalence of high-risk HPV, specifically HPV35, 45, 52, 56, 59, and 68, was significantly increased from 2015 to 2021 (all *p *< 0.01); however, substantial decreasing trends were found for HPV33 and HPV66 (both *p* < 0.01; Table 2).

**Table 2.** Prevalence of HPV types in Guangzhou China by year.

HPV type	2015	2016	2017	2018	2019	2020	2021	gamma value	*p*	Trend
Number of cases	26055	27965	30620	30972	31328	22730	28441	–	–	
Positive cases	5415 (20.78)	5515 (19.72)	6285 (20.53)	6941 (22.41)	6833 (21.81)	5539 (24.37)	6383 (22.44)	0.042	<0.001	Increasing
Positive of low-risk	1146 (4.40)	1285 (4.60)	1495 (4.88)	1852 (5.98)	1746 (5.57)	1568 (6.90)	1734 (6.10)	0.091	<0.001	Increasing
Positive of high-risk	4825 (18.52)	4821 (17.24)	5486 (17.92)	5896 (19.04)	5847 (18.66)	4628 (20.36)	5407 (19.01)	0.025	<0.001	Increasing
HPV16	825 (3.17)	788 (2.82)	882 (2.88)	884 (2.85)	839 (2.68)	712 (3.13)	777 (2.73)	−0.016	0.073	
HPV18	320 (1.23)	314 (1.12)	359 (1.17)	386 (1.25)	348 (1.11)	265 (1.17)	339 (1.19)	−0.003	0.813	
HPV31	161 (0.62)	173 (0.62)	188 (0.61)	199 (0.64)	186 (0.59)	143 (0.63)	173 (0.61)	−0.003	0.881	
HPV33	233 (0.89)	270 (0.97)	290 (0.95)	244 (0.79)	267 (0.85)	188 (0.83)	198 (0.70)	−0.055	0.001	Decreasing
HPV35	50 (0.19)	52 (0.19)	90 (0.29)	115 (0.37)	96 (0.31)	89 (0.39)	97 (0.34)	0.134	<0.001	Increasing
HPV39	504 (1.93)	519 (1.86)	527 (1.72)	602 (1.94)	572 (1.83)	462 (2.03)	539 (1.90)	0.008	0.464	
HPV45	60 (0.23)	67 (0.24)	103 (0.34)	75 (0.24)	88 (0.28)	80 (0.35)	97 (0.34)	0.077	0.006	Increasing
HPV51	497 (1.91)	490 (1.75)	606 (1.98)	587 (1.90)	618 (1.97)	477 (2.10)	541 (1.90)	0.016	0.146	
HPV52	1403 (5.38)	1410 (5.04)	1468 (4.79)	1802 (5.82)	1852 (5.91)	1455 (6.40)	1703 (5.99)	0.050	<0.001	Increasing
HPV53	535 (2.05)	564 (2.02)	528 (1.72)	599 (1.93)	533 (1.70)	454 (2.00)	548 (1.93)	−0.012	0.277	
HPV56	160 (0.61)	169 (0.60)	268 (0.88)	326 (1.05)	325 (1.04)	290 (1.28)	304 (1.07)	0.143	<0.001	Increasing
HPV58	734 (2.82)	648 (2.32)	769 (2.51)	828 (2.67)	775 (2.47)	641 (2.82)	737 (2.59)	0.005	0.601	
HPV59	107 (0.41)	119 (0.43)	196 (0.64)	216 (0.70)	215 (0.69)	184 (0.81)	208 (0.73)	0.130	<0.001	Increasing
HPV66	235 (0.90)	259 (0.93)	285 (0.93)	232 (0.75)	240 (0.77)	181 (0.80)	226 (0.79)	−0.044	0.008	Decreasing
HPV68	330 (1.27)	353 (1.26)	470 (1.53)	574 (1.85)	478 (1.53)	368 (1.62)	489 (1.72)	0.063	<0.001	Increasing

HPV, human papillomavirus.

### HPV prevalence with the progressive cancer

**Figure 2.**
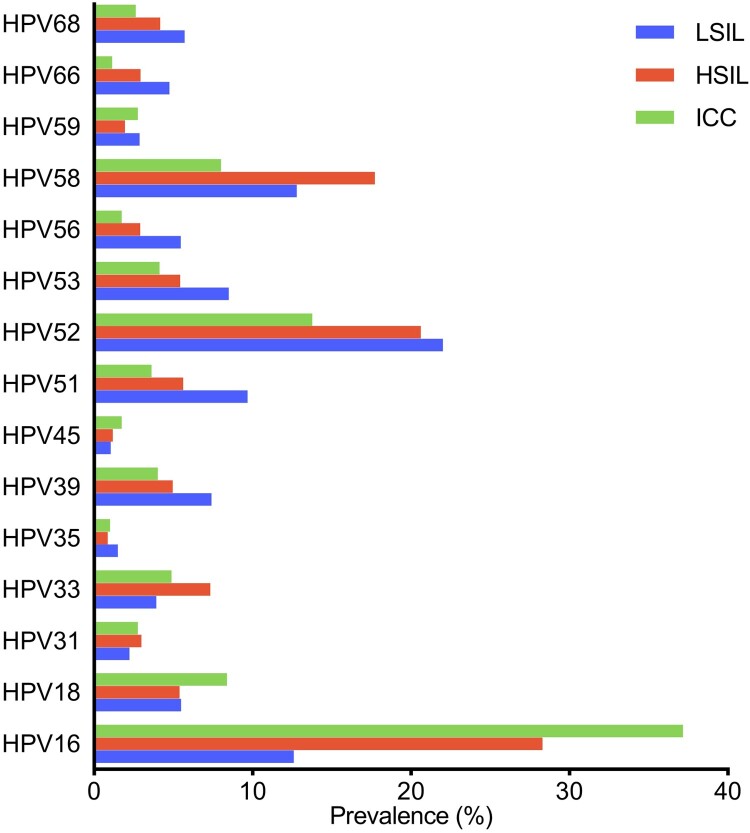
High-risk HPV prevalence associated with the severity of intraepithelial lesions in Guangzhou, China. HPV, human papillomavirus; LSIL, low-grade squamous lesion; HSIL, high-grade squamous lesion; ICC, invasive cervical carcinoma.

With the development of cervical carcinogenesis, the HPV prevalence significantly increased, from 78.91% in LSIL to 84.48% in ICC (*p* < 0.001). Regarding the types, HPV16 showed a significant increase with the progressive cancer, from 12.60% in patients with LSIL to 37.17% in those with ICC (*p* < 0.001), as did HPV31, HPV33, and HPV58. In contrast, the prevalence of HPV 52 decreased from 22.02% in patients with LSILs to 13.77% in patients with ICC (*p* < 0.001), which is in line with the trends observed for other HPV, including HPV35, 39, 51, 53, 59, 66, and 68 (Figure 2; Supplementary Table S1). Regardless, HPV52 was the second-most observed type in ICC (Table 1). Moreover, the HPV18 prevalence in ICC was significantly higher than that in LSIL (8.39% vs. 5.48%, *p* < 0.01) and HSIL (8.39% vs. 5.40%, *p* < 0.01), but no significant difference was found between LSIL and HSIL (*p* > 0.05).

### Single and multiple HPV infections

**Figure 3.**
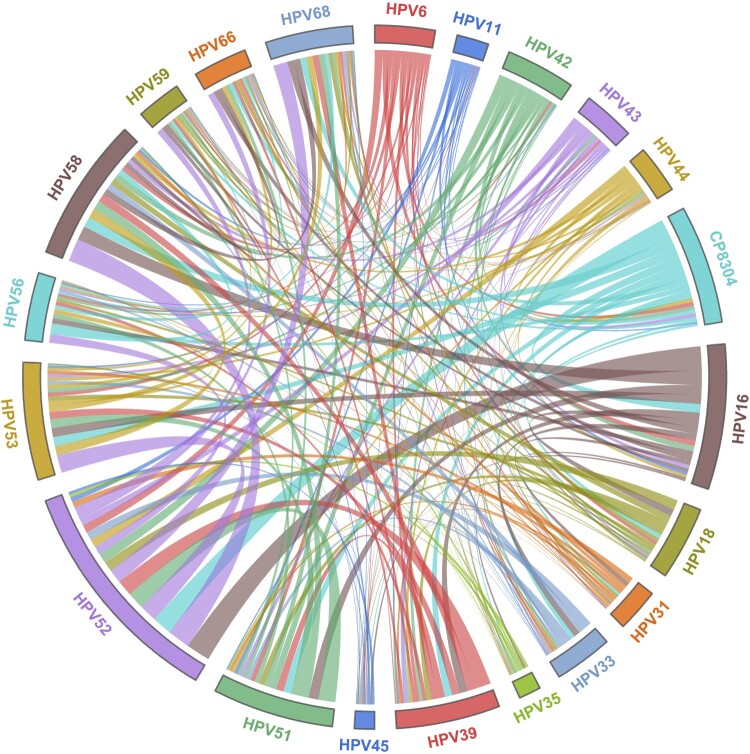
Chord diagram of HPV type correlations in women with multiple infections in Guangzhou, China. HPV, human papillomavirus.

Among 42,911 HPV infections, 73.49% (31536/42911) were caused by a single HPV type, especially one high-risk type (60.22%, 25841/42911). Regarding the 26.51% (11375/42911) of multiple infections, 18.83% comprised double infections, 5.24% were triple infections, 1.67% comprised quadruple infections, and 0.77% were quintuple infections or more. With the development of cervical lesions, a decreasing trend in the prevalence of multiple infections was found from LSIL (38.72%) to HSIL (33.49%) to ICC (22.52%) (*p* < 0.001; Table 3). Additionally, pairwise combinations of different HPV types in co-infections were clearly observed in the chord plot (Figure 3), and the most common combinations were HPV16 and HPV52 (705 times), HPV52 and HPV58 (663 times), and HPV52 and CP8304 (570 times). However, for patients with CA, the rates of a single low-risk infection and multiple infections were significantly higher than those in other groups (*p* < 0.05; Table 3).

**Table 3.** Single and multiple infection status in 42911 females with HPV infection.

HPV infection	Normal cervix	Inflammation	ASC-US/-H	LSIL	HSIL	ICC	CA	Others
Number of cases	8557 (19.94)	17066 (39.77)	5952 (13.87)	6398 (14.91)	2726 (6.35)	675 (1.57)	423 (0.99)	1114 (2.6)
Single infection	6573 (76.81)	13423 (78.65)**	4162 (69.93)***	3921 (61.28)***	1813 (66.51)***	523 (77.48)	237 (56.03)***	884 (79.35)
Single low-risk infection	1340 (15.66)	2835 (16.61)	595 (10.00)***	427 (6.67)***	128 (4.70)***	57 (8.44) ***	112 (26.48)***	201 (18.04)***
Single high-risk infection	5233 (61.15)	10588 (62.04)	3567 (59.93)	3494 (54.61)***	1685 (61.81)	466 (69.04)***	125 (29.55)***	683 (61.31)
Multiple infection	1984 (23.19)	3643 (21.35)**	1790 (30.07)***	2477 (38.72)***	913 (33.49)***	152 (22.52)	186 (43.97)***	230 (20.65)
Double infection	1479 (17.28)	2778 (16.28)*	1270 (21.34)***	1590 (24.85)***	582 (21.35)***	114 (16.89)	108 (25.53)***	160 (14.36)*
Triple infection	360 (4.21)	621 (3.64)*	356 (5.98)***	570 (8.91)***	224 (8.22)***	21 (3.11)	46 (10.87)***	49 (4.4)
Quadruple infection	100 (1.17)	187 (1.1)	111 (1.86)**	206 (3.22)***	68 (2.49)***	11 (1.63)	16 (3.78)***	16 (1.44)
Quintuple infection or more	45 (0.53)	57 (0.33)*	53 (0.89)**	111 (1.73)***	39 (1.43)***	6 (0.89)	16 (3.78)***	5 (0.45)

HPV, human papillomavirus; ASC-US/-H, atypical squamous cells of undetermined significance or cannot excluded HSIL; LSIL, low-grade squamous lesion; HSIL, high-grade squamous lesion; ICC, invasive cervical carcinoma; CA, condyloma acuminate.

Normal cervix is served as test control.

***The significance is at the *p* < 0.001 level.

**The significance is at the *p* < 0.01 level.

*The significance is at the *p* < 0.05 level.

### Age-specific HPV prevalence

**Figure 4.**
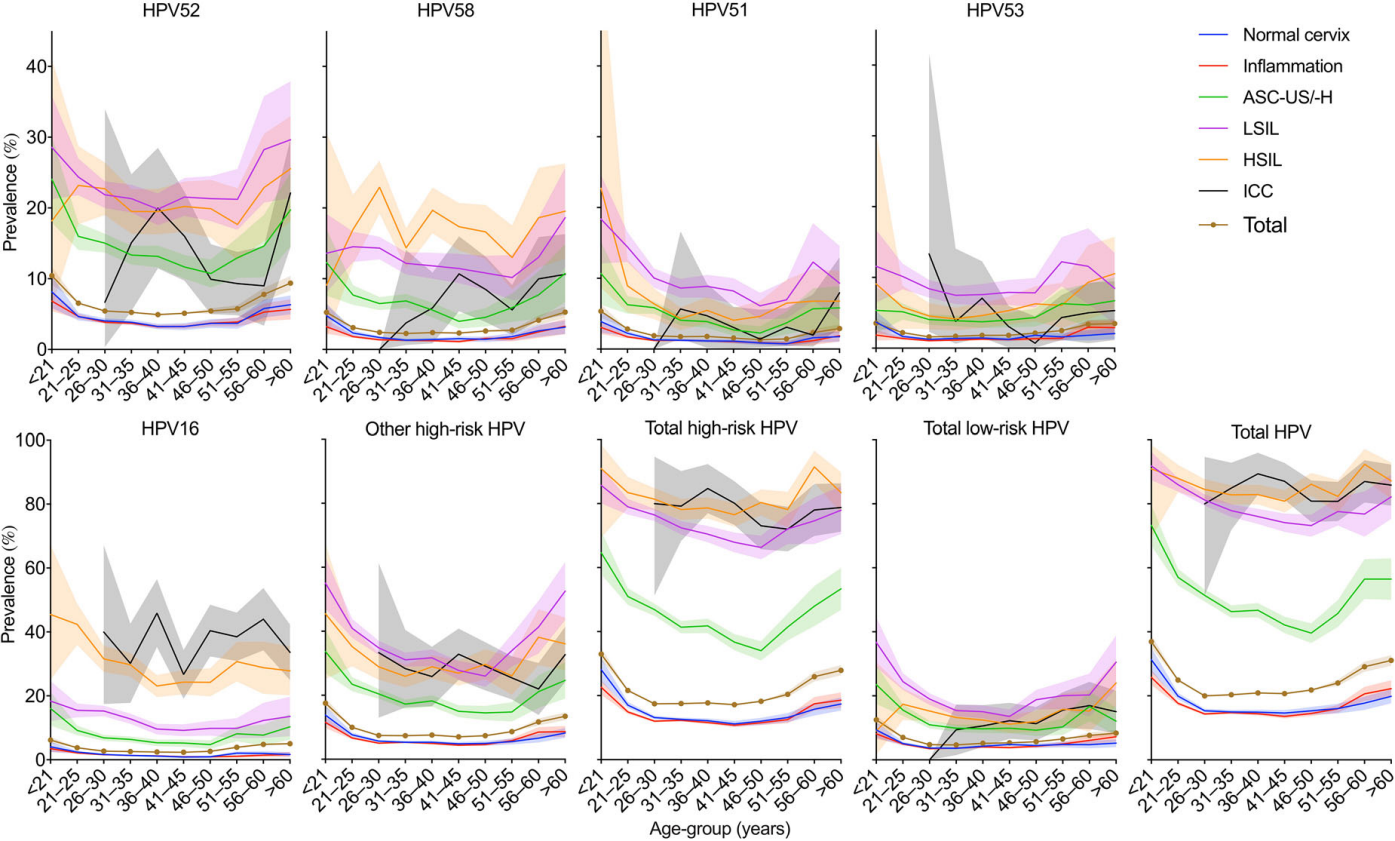
Age-specific HPV prevalence among women in Guangzhou, China, according to diagnosis. HPV, human papillomavirus; LSIL, low-grade squamous lesion; HSIL, high-grade squamous lesion; ICC, invasive cervical carcinoma; CA, condyloma acuminate, ASC-US/-H, atypical squamous cells of undetermined significance or HSIL not excluded. Shaded areas represent 95% CIs.

Figure 4 shows the changes of HPV prevalence by age in different diagnoses. The prevalence was highest in females aged < 21 years and then decreased rapidly; this did not progressively increase from 31 to 45 years of age. In the 46–50-year age group, the prevalence increased among all HPV types and reached a second peak. For the normal cervix and inflammation groups, the prevalence of all types gradually decreased from the highest point in the < 21-year group to the lowest point in the 26–30-year group; it was then relatively stable from 31 to 55 years of age and increased in the 55–60-year or older age group. For the groups of ASC-US/-H, LSIL, and HSIL, the positive rate decreased continuously with increasing age until the 41–45-year or older age group, when the prevalence increased among all groups, except in HPV58-associated HSIL, for which the rates continued to increase up to 30 years of age. However, the total prevalence in the ICC group varied between approximately 80% and 90% for all age groups.

### Time to HPV clearance

**Figure 5.**
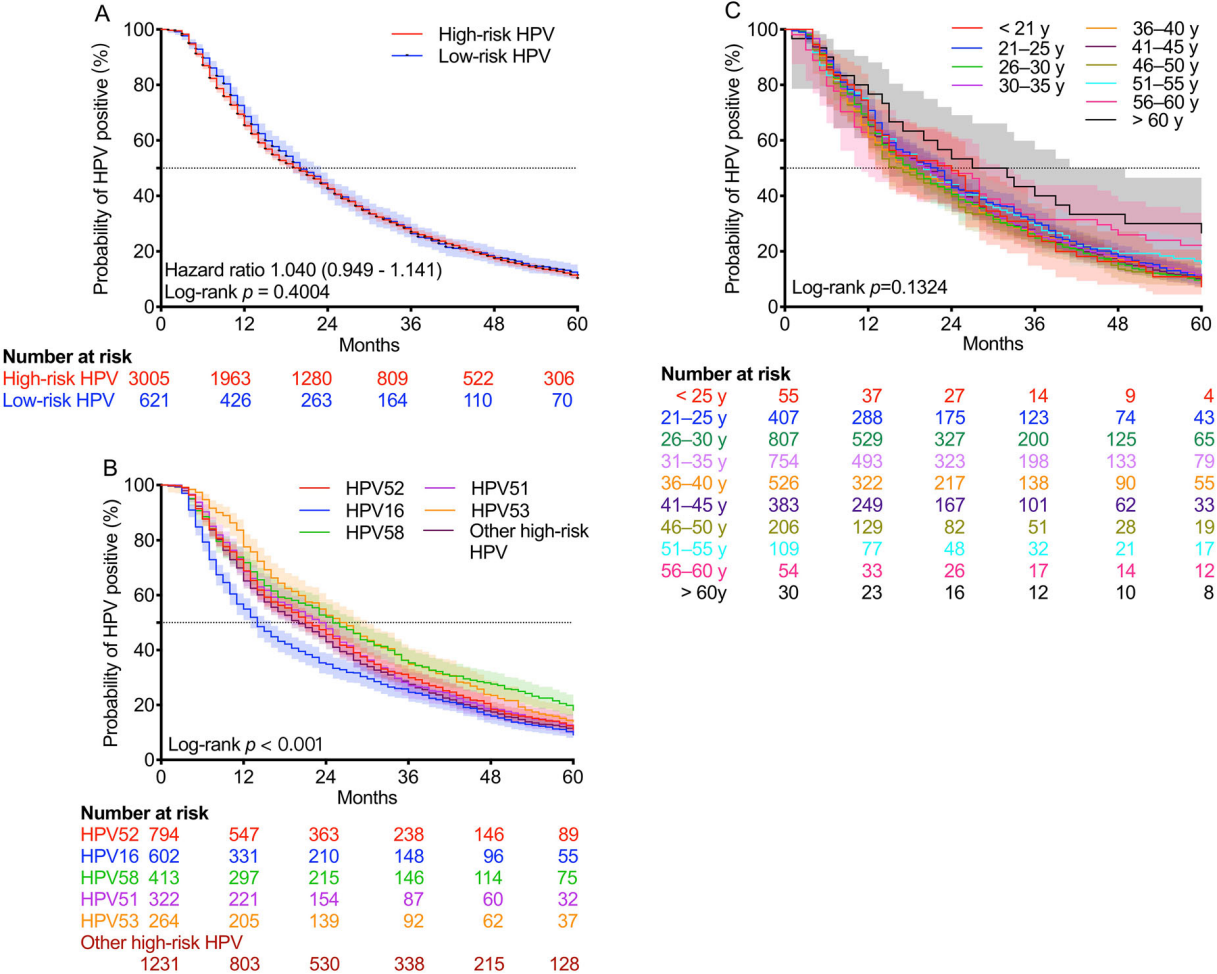
Time to clearance of HPV infection in HPV-positive women during long-term monitoring. HPV, human papillomavirus. Shaded areas represent 95% CIs.

Continuous monitoring was performed for 3331 HPV-positive women within the subsequent 5 years. The median time to HPV clearance was 16 (9–31) months, and the viral clearance rate within 5 years was 89.94% (2996/3331). The median times to HPV clearance of high-risk and low-risk were 16 (9–31) and 18 (10–31) months, and the viral clearance rates within 5 years were 89.82% (2699/3005) and 88.73% (551/621), respectively. No significant difference was found between women infected with high-risk and low-risk HPV (HR = 1.040, *p* > 0.05; Figure 5 (A)).

The median times to viral clearance in patients infected with HPV16, 51, 52, 53, 58, and other high-risk types were 14 (7–36), 24 (11–40), 22 (11–42), 27 (14–47), 25 (11–52), and 20 (10–39) months, respectively, and the viral clearance rates within 5 years were 90.86% (547/602), 90.06% (290/322), 88.79% (705/794), 85.98% (227/264), 81.84% (338/413), and 89.60% (1103/1231), respectively (Figure 5 (B)). Of note, viral clearance of HPV16 was significantly faster than that of other HPV types (*p* < 0.001). The median times to viral clearance in females aged < 21, 21-25, 26-30, 31-35, 36-40, 41-45, 46-50, 51-55, 56-60, and > 60 years old were 24 (11.5–36), 22 (11–40), 18 (10–36), 21 (10–38), 18 (9–38), 21 (10–39), 17 (9–36), 20 (12–41), 24 (8–51), and 30 (14–60) months, respectively, and the viral clearance rates within 5 years were 92.73% (51/55), 89.43% (364/407), 91.95% (742/807), 89.52% (675/754), 89.54% (471/526), 91.38% (350/383), 90.78% (187/206), 84.40% (92/109), 77.78% (42/54), and 73.33% (22/30), respectively (Figure 5 (C)). There were no significant differences among these groups (*p* > 0.05).

## Discussion

This large, long-term study of 198,111 women in Guangzhou, China, produced the most precise and representative data of HPV burden in south China to date. Our analysis shows that the overall prevalence of HPV was 21.66%, and the annual prevalence increased significantly from 2015 to 2021. HPV52, 16, 58, CP8304, 51, 53, 39, and 68 were the most common types. HPV prevalence was found to increase with the development of intraepithelial lesions, with three of the most prominent strains being HPV16, 18, and 52. Our research revealed that HPV prevalence was significantly age-specific, and two clear peaks were observed in women < 21 years old and > 46 years old, respectively. Notably, the median time to HPV clearance was 16 (9–31) months, and the viral clearance rate within 5 years was 89.94%.

HPV prevalence varies greatly in different regions of the world. In this study, the HPV prevalence over the past 7 years was 21.66%, which was consistent with the 21.07% (ranging from 18.42% to 31.94%) previously reported in 37 cities in China [21]. As the symptoms of most HPV infections are not obvious, early detection of HPV is critical for CC prevention. This study revealed an HPV prevalence of 16.01% among the 53,439 females with a normal cervix, which was similar to the 15.63% reported in the neighboring city of Shenzhen [22]. However, when examined by region, different rates of HPV prevalence in women with a normal cervix were reported, including 7.3–21.3% in other regions of China [17,20,23,24], 8.1% in Europe, 13.0% in the Americas, and 22.1% in Africa [16], greatly affected by demographic factors, socioeconomic status, education level, poor cervical screening, and vaccination programs [16,25]. More refined strategies to reduce infection rate and potentially reduce risks for developing cervical carcinogenesis based on the uneven distribution of HPV incidence are needed.

As the main cause of cervical malignancy, HPV can be found in almost all CCs tested under ideal conditions [26]. This analysis confirmed that HPV infection significantly increases with intraepithelial lesion development, with a prevalence of up to 84.48% in ICC patients, similar to that in previously reported studies in neighboring Shenzhen (88.57%) [22], Eastern Qingdao (90.4%) [27], Western Shaanxi (78.5) [23], and Northern Urumqi (100%) [28], but significantly higher than that in the country’s capital of Beijing (34.6%) [29]. Regarding the types, HPV16 is the most frequent type with a prevalence of 28.31% and 37.17% among patients with HSIL and ICC, respectively. Comparatively, as the second-most frequent carcinogenic strain worldwide, the ranking of HPV18 prevalence varies by region. In this study, it only ranked ninth (affecting only 1.18% of the study sample and 0.70% of women with a normal cytology), consistent with the low prevalence reported in several Chinese studies (0.70–1.56%) [22,24,29], but inconsistent with a meta-analysis showing that HPV18 is the second-most observed HPV type among females with normal cytology in Eastern Asia and Southeastern Asia [16]. Of note, HPV18 prevalence significantly increased from 1.18% in individuals with a normal cervix to 5.48% in LSIL and HPV18 was the third-most common type, with a prevalence of 8.39%, in ICC. Compared with that of other high-risk types, the carcinogenicity of HPV16 and HPV18 can lead to cervical lesions; more than 50–80% of severe cervical lesions were infected by HPV16 or HPV18 in previous studies [30–32]. Although much rarer, HPV31, HPV33, and HPV58 might also have this oncogenic advantage as they are positively correlated with CC development. More importantly, previous surveys showed that HPV33 and HPV58 have become the most carcinogenic types in some regions [14,17]. These observations will help guide the future HPV vaccine development strategies against cervical carcinogenesis.

Similar to that in other regions in China such as Beijing (5.64%) [29], Sichuan (7.01%) [17], and Wuhan (4.23%) [33], HPV52 was the most observed type detected in Guangzhou, with a rate of 5.60%. However, this was not consistent with one study that reported an HPV52 prevalence of 0.9% (ranked fourth) among women with a normal cytology in Eastern Asia (excluding Japan and Taiwan) [16]. Notwithstanding, a significant decrease was found in HPV52 positivity with the development of cervical lesions (from 22.02% in LSIL to 13.77% in ICC, *p *< 0.001; Supplementary Table S1). The higher positivity rate in ICC compared with the rate of 4.02% reported in individuals with a normal cervix also seems to confirm that HPV52 is the most carcinogenic type only after HPV16. When excluding co-infection with HPV16 or HPV18, HPV52-associated ICC was significantly less common than lesions associated with HPV16 (0.82% vs. 5.55%, *p *< 0.001) and HPV18 (0.82% vs. 2.97%, *p *< 0.001) (Supplementary Figure S1). These observations reveal that compared with HPV16 and HPV18, the advantage of HPV52 in terms of persistence and progression to CC needs further investigation.

Most notably, the prevalence of HPV52 increased by year, ranging from 5.04% in 2016–6.40% in 2020, whereas no change in prevalence was found for both HPV16 and 58. However, previous studies have shown that HPV52 was the second-most frequent type after HPV16 in this region before 2014 [14,21]. This result is potentially significant because it suggests that HPV52 has become the dominant type in Guangzhou, a situation that is still developing.

Consistent with the results of previous studies [15,16,21], our study also showed a “two-peak” pattern of age-specific prevalence in Guangzhou. The first peak was found in young females (< 21 years old), with the highest rate of 36.97%. Because of immature immune protection, the prevalence of young females can reach up to 80% in some populations, soon after beginning sexual activity [34]. Crucially, the relatively low vaccine coverage of 3.09% in young females in this region greatly increases the prevalence of HPV [35]. Then, the HPV infection rate gradually declined to a plateau until the age of 46 years, after which it reached a second peak. Globally, a second peak was observed in those older than 44 years in the Americas, in those nearly 50 years in China, and in those 45 years or older in Europe and Africa [16,21]. The age-specific second peak observed could be associated with immunosenescence caused by hormonal changes in climacteric women [16], new sexual partners in their middle age [36], and viral characteristics [37]. Nonetheless, the exact mechanisms underlying this “two-peak” pattern still remain unclear.

Usually, most HPV is rapidly eliminated by immunological intervention after viral infection [38], and only 10% of patients experience persistent infection [39]. Longer HPV persistence increases the risk of a cancer diagnosis [40]. Our clearance estimates showed that 89.94% of HPV infections were eliminated within 5 years, and the median clearance time was 16 (9–31) months. During screening studies, the time to clearance for HPV infections was previously found to be 6–18 months [40]. Additionally, previous data showed that HPV infections are typically cleared rapidly, with 67% being cleared by 12 months [41]. Prevalent infections persisted longer in our study probably because many patients were not followed up regularly after HPV clearance and the patients included in follow-up studies tended to have a persistent HPV infection or accompanying cytopathic signs. Moreover, early clearance often occurs faster than it can be measured, and the long-detection interval from a positive result to a negative result on subsequent testing may also the reasons.

Our results revealed that the clearance of HPV16 occurs faster than that of other types. This phenomenon is compatible with the potentially increased awareness of HPV16-associated cancer in females and the fact that these infections are more likely to receive timely and standardized treatment than other types. However, another study showed that the clearance time of some other types can be prolonged [42]. As older women are more likely to have long-term infections, HPV in these women will persist longer than that in younger. Interestingly, the persistence of these prevalent HPV showed no significant differences among different age groups in the present study.

Finally, CA refers to benign hypertrophic lesions in anal/genital area, attributed mostly to low-risk HPV types, which are frequently present as co-infections with high-risk HPV. In our analysis, most CA patients were infected by low-risk HPV6 and HPV11, and 7.52% were infected by a single strain or co-infected with HPV52. This observation is concordant with those in other prospective studies [43,44].

Our work has produced some convincing results, but there are still several limitations. First, this study is a single-center, hospital-based, and retrospective study. Second, we were unable to reliably track whether those females received additional therapies or medication treatment in other hospital after the initial visit. The HPV clearance was evaluated in females with and without treatment or surgery. Third, as some behaviors (e.g. smoking, sexual activity, dietary patterns) may be associated with infection of HPV [45,46]. Further research about the correlation between behavioral information and HPV infection needs to be performed.

## Conclusions

In summary, this study showed an overall HPV prevalence of 21.66% (42911/198111), with the annual rate significantly increasing from 2015 to 2021 in Guangzhou, China. HPV52, 16, 58, CP8304, 51, 53, 39, and 68 were the most prevalent HPV types. HPV16 was the predominant carcinogenic type, followed by HPV52 and HPV18. HPV infections were significantly age-specific, and 26.51% (11,375/42,911) of cases were caused by multiple HPV types. HPV infections were typically cleared with a median time of 16 months. These findings provide a valuable guide for local governments to evaluate cervical screening and vaccination programs in south China.

## Supplementary Material

Supplementary_materials.doc

## Data Availability

The data that support the findings of this study are available from the corresponding author upon reasonable request.
