# Supplementary material for: Cervical HPV infection in Guangzhou, China: an epidemiological study of 198,111 women from 2015 to 2021
Source: Emerg Microbes Infect. 2023 Feb 15;12(1):e2176009. doi: 10.1080/22221751.2023.2176009 (PMC9936994; doi:10.1080/22221751.2023.2176009)
Supplement: Supplementary_materials.doc [file TEMI_A_2176009_SM4430.doc]

Table S1. Relative distribution of HPV genotypes according to cervical lesion status

| HPV type | LSIL | HSIL | ICC | garmma | *p*^a^ | Trend |
| --- | --- | --- | --- | --- | --- | --- |
| No. of cases | 8108 | 3243 | 799 | - | - |  |
| Positive of HPV | 6398 (78.91) | 2726 (84.06) | 675 (84.48) | 0.163 | <0.001 | Increasing |
| Positive of high-risk | 5942 (73.29) | 2591 (79.90) | 617 (77.22) | 0.151 | <0.001 | Increasing |
| Positive of low-risk | 1485 (18.32) | 451 (13.91) | 105 (13.14) | -0.160 | <0.001 | Decreasing |
| HPV16 | 1022 (12.60) | 918 (28.31) | 297 (37.17) | 0.470 | <0.001 | Increasing |
| HPV18 | 444 (5.48) | 175 (5.40) | 67 (8.39) | 0.063 | 0.107 |  |
| HPV31 | 181 (2.23) | 97 (2.99) | 22 (2.75) | 0.126 | 0.031 | Increasing |
| HPV33 | 319 (3.93) | 238 (7.34) | 39 (4.88) | 0.244 | <0.001 | Increasing |
| HPV35 | 122 (1.50) | 28 (0.86) | 8 (1.00) | -0.240 | 0.003 | Decreasing |
| HPV39 | 600 (7.40) | 161 (4.96) | 32 (4.01) | -0.222 | <0.001 | Decreasing |
| HPV45 | 85 (1.05) | 38 (1.17) | 14 (1.75) | 0.112 | 0.189 |  |
| HPV51 | 785 (9.68) | 182 (5.61) | 29 (3.63) | -0.317 | <0.001 | Decreasing |
| HPV52 | 1785 (22.02) | 669 (20.63) | 110 (13.77) | -0.094 | <0.001 | Decreasing |
| HPV53 | 688 (8.49) | 176 (5.43) | 33 (4.13) | -0.253 | <0.001 | Decreasing |
| HPV56 | 443 (5.46) | 94 (2.90) | 14 (1.75) | -0.351 | <0.001 | Decreasing |
| HPV58 | 1037 (12.79) | 575 (17.73) | 64 (8.01) | 0.085 | 0.001 | Increasing |
| HPV59 | 233 (2.87) | 63 (1.94) | 22 (2.75) | -0.135 | 0.017 | Decreasing |
| HPV66 | 386 (4.76) | 95 (2.93) | 9 (1.13) | -0.315 | <0.001 | Decreasing |
| HPV68 | 463 (5.71) | 135 (4.16) | 21 (2.63) | -0.204 | <0.001 | Decreasing |

HPV, human papillomavirus; LSIL, low-grade squamous intraepithelial lesion; HSIL, high-grade squamous intraepithelial lesion; ICC, invasive cervical cancer.

^a^Linear‐by‐linear association test.

Figure S1. Prevalence of HPV16, 18 and 52 associated with the severity of intraepithelial lesions in Guangzhou, China. HPV, human papillomavirus; LSIL, low-grade squamous lesion; HSIL, high-grade squamous lesion; ICC, invasive cervical carcinoma; CA, condyloma acuminate, ASC-US/-H, atypical squamous cells of undetermined significance or HSIL not excluded.
